# Revisit ^18^F-fluorodeoxyglucose oncology positron emission tomography: “systems molecular imaging” of glucose metabolism

**DOI:** 10.18632/oncotarget.16647

**Published:** 2017-03-29

**Authors:** Baozhong Shen, Tao Huang, Yingying Sun, Zhongnan Jin, Xiao-Feng Li

**Affiliations:** ^1^ PET/CT/MRI Center, The Fourth Hospital of Harbin Medical University, Harbin, China; ^2^ Molecular Imaging Research Center, Harbin Medical University, Harbin, China; ^3^ Department of Radiology, The Fourth Hospital of Harbin Medical University, Harbin, China

**Keywords:** ^18^F-FDG, positron emission tomography, cancer, hypoxia, glucose metabolism

## Abstract

^18^F-fluorodeoxyglucose (^18^F-FDG) positron emission tomography has become an important tool for detection, staging and management of many types of cancer. Oncology application of ^18^F-FDG bases on the knowledge that increase in glucose demand and utilization is a fundamental features of cancer. Pasteur effect, Warburg effect and reverse Warburg effect have been used to explain glucose metabolism in cancer. ^18^F-FDG accumulation in cancer is reportedly microenvironment-dependent, ^18^F-FDG avidly accumulates in poorly proliferating and hypoxic cancer cells, but low in well perfused (and proliferating) cancer cells. Cancer is a heterogeneous and complex “organ” containing multiple components, therefore, cancer needs to be investigated from systems biology point of view, we proposed the concept of “systems molecular imaging” for much better understanding systems biology of cancer.

This article revisits ^18^F-FDG uptake mechanisms, its oncology applications and the role of ^18^F-FDG PET for “systems molecular imaging”.

## INTRODUCTION

^18^F-fluorodeoxyglucose (^18^F-FDG) positron emission tomography (PET) imaging has emerged as an important clinical tool for cancer detection, staging and monitoring of response to therapy, ^18^F-FDG PET is routinely used in the clinical management of several cancer types [1].

^18^F-FDG, a glucose analog, was synthesized approximate 4 decades ago. In 1968, Pacak et al were the first to describe the synthesis of “cold” FDG [2]. In the 1970s, Ido and Wolf reported the synthesis of ^18^F-FDG [3]. In 1976, researchers at the University of Pennsylvania obtained the first ^18^F-FDG brain images in two health volunteers [4].

In 1861, French chemist and microbiologist Louis Pasteur found the increase in cell mass of yeast under anaerobic condition was smaller than aerobically, but the rate of sugar breakdown in yeast was higher in anerobic condition than the presence of oxygen, referred as Pasteur effect [5–7]. Pasteur effect can be easily explained when the oxygen concentration is low, the energy production efficiency is low. If the oxygen concentration grows, yeast increases the efficiency to 32 moles of adenosine triphosphate (ATP) per mole of glucose. Although Pasteur effect seems more frequently apply in microbiology, it oncology application is less popular. In fact, ^18^F-FDG accumulation is found significant higher in hypoxic cancer cells than in well oxygenated ones, which may be explained by Pasteur effect [8–12].

In early 1920s, German biologist Otto Warburg reported that, in the presence of ample oxygen, cancer cells prefer to metabolize glucose by “aerobic glycolysis”, so called Warburg effect [13]. The increase in glucose demand has been considered as one of the fundamental features of cancer [13, 14], which has also become the fundamental of ^18^F-FDG PET in oncology application. However, some malignancies had low ^18^F-FDG PET findings [15], which is unexplainable by Warburg effect. Therefore, better understanding of ^18^F-FDG uptake mechanism is important for better cancer practice.

In 2009, Lisanti and colleagues proposed “the reverse Warburg effect” hypothesis in human breast cancers. They claimed the Warburg effect actually took place in tumor associated fibroblasts, and not in cancer cells [16–20]. Zhang et al have examined the hypothesis in cancer cells growing in nude mice with ^18^F-FDG PET study [8].

Understanding ^18^F-FDG uptake mechanism in cancer is essential for better understanding cancer biology. Cancer is not a collection of pure cancer cells, which is composed by multiple complex components and is considered as an “organ”, the so called systems biology. In addition, cancer also follows “Darwin Dynamics” for survival, in other words, the fraction of each component or molecular level events of cancer may change dramatically to adapt the change of microenvironment [21].

In this article, we revisited the ^18^F-FDG uptake mechanisms and its oncology applications. We propose “systems molecular imaging” initiative for better understanding systems biology of cancer, and we discussed the role of ^18^F-FDG PET in systems molecular imaging.

## RESULTS AND DISCUSSION

### ^18^F-FDG uptake mechanism in cancer

#### Highly heterogeneous ^18^F-FDG uptake in viable cancer cells is unlikely explained by Warburg effect

Warburg reported that even in the presence of ample oxygen, cancer cells prefer to metabolize glucose by “aerobic glycolysis” because of mitochondrial dysfunction in cancer cells; so called Warburg effect [13]. Accordingly, the existence of aerobic glycolysis would confer an uniformed increase in ^18^F-FDG uptake in all viable cancer cells within tumors and unrelated to cancer cell oxygen status. Growing evidence indicated that intratumoral ^18^F-FDG distribution is heterogeneous and microenvironment dependent. Hypoxic cancer cells had significantly higher ^18^F-FDG uptake than oxic cancer cells in *in vitro* studies [22–25] and *in vivo* animal studies [8–12, 26, 27], hence, ^18^F-FDG accumulation in cancer can not be reasonably explained by Warburg effect.

#### ^18^F-FDG uptake and tumor hypoxia: the critical role of pasteur effect

During the past decade, investigators had demonstrated in cancer cell culture and animal models of cancer that hypoxic condition played a critical role inducing high ^18^F-FDG accumulation. Clavo et al [22] showed that FDG uptake in melanoma and ovarian carcinoma cell lines was increased under hypoxic conditions in O_2_ concentration-dependent manner. In addition, Burgman et al observed hypoxia dependent FDG uptakes in breast cancer cells [23]. Furthermore, intratumoral distribution of ^18^F-FDG in cancer models growing in animals colocalized with pimonidazole labeled cancer cells [8–12].

**Figure 1:**
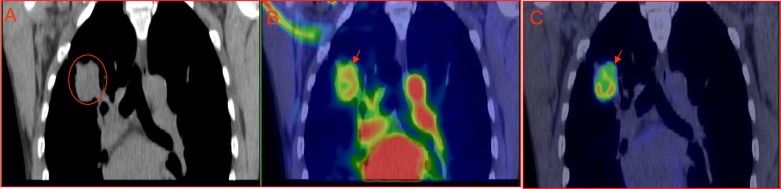
Mismatched intratumoral distribution between early phase perfusion imaging ***vs*** 60-min metabolism imaging. A 52-yr-old male with pathologically confirmed right lung adenocarcinoma, the patient is given 6 mCi (222MBq) ^18^F-FDG bolus injection and thoracic PET/CT scan starts simultaneously lasting for 5 min (early phase perfusion imaging), the patient has whole body PET/CT scan 60 minutes after injection. There is apparent mismatched between perfusion and metabolism. A: CT imaging; B: perfusion^18^F-FDG PET/CT overlay (0-5min); C: metabolism^18^F-FDG PET/CT imaging.

Spatial co-localization was found between high ^18^F-FDG uptake and tumor hypoxia, and such regions also had low blood perfusion. On the other hand, non-hypoxic regions displayed low ^18^F-FDG uptake. In addition, both stroma and necrotic zones were also associated with lower ^18^F-FDG activity [11]. Our clinical data support the preclinical findings, high glucose metabolism region at 60-min ^18^F-FDG PET of lung cancer associated with low blood perfusion detected by early phase ^18^F-FDG perfusion imaging. However, high blood perfusion region at early phase ^18^F-FDG perfusion associated with low glucose metabolism region 60-min after ^18^F-FDG bolus injection (Figure 1). Beer et al demonstrated intratumoral distribution of ^18^F-FDG and ^18^F-galacto-RGD in patients was mismatched; ^18^F-galacto-RGD is predominantly α_v_β_3_ avid which are expressed in endothelial cells of new blood vessels [28]. These findings clearly demonstrated that viable and well perfused cancer regions in patients were associated with low ^18^F-FDG uptake, while as regions with high ^18^F-FDG accumulation had low to null blood perfusion. Therefore, ^18^F-FDG uptake in cancer can be perfectly explained by Pasteur effect, but not Warburg effect.

^18^F-FDG uptake in non-hypoxic (oxic) cancer cells is low [8–12]; therefore, ^18^F-FDG can not map oxic cancer cells. This finding is critically important, especially for assessing anticancer effect with ^18^F-FDG PET/CT; a negative ^18^F-FDG PET finding does not necessarily mean the absence of viable cancer cells, which simply indicates the absence of hypoxic cells.

Of note, although oxic cancer cells have low glucose demand than hypoxic cancer cells, which does not mean oxic cancer cells use less energy for their biological processes. In contrast, since oxic cells are more proliferative than hypoxic cancer cells, oxic cancer cells must use more ATP than hypoxic cells to repopulate. A reasonable explanation is that in the presence of oxygen, cancer cells may utilize glucose to generate energy by switching in a more efficient oxidative phosphorylation glucose metabolism pathway which requires significantly less glucose to generate greater amount of ATP.

#### ^18^F-FDG uptake and reverse Warburg effect

With respect to reverse Warburg effect hypothesis [16–20], Zhang et al found, in an animal model of non-small cell lung cancer, that ^18^F-FDG accumulated in the stroma of solid tumors rather than cancer cells only in case of those study when animals were under after meal condition [8]. Considering that all patients are required to be fasted at least 6 hours before ^18^F-FDG PET study, it is unlikely reverse Warburg effect is ideal to explain ^18^F-FDG uptake in solid malignancies, the guidance of reverse Warburg effect for understanding ^18^F-FDG PET imaging may have less clinical significance.

#### ^18^F-FDG uptake and proliferation

Macroscopic solid cancers have a complex microenvironment with intermingled but clearly defined regions of non-cancerous stroma, areas of cancer cells and necrosis, which systems cancer biology focuses on. Cancer cells may further divide into two subcategories according to oxygenation status and cellular proliferation: Cancer cells closely adjacent to functional blood vessels are well-oxygenated with higher proliferation rate. Hypoxic cancer cells generally locate 100-200 μm away from functional blood vessels and/or close to areas of necrosis with a low proliferation rate [29]. Although cellular proliferation and hypoxia are generally exclusive, the presence of tumor hypoxia is because of faster cancer cells proliferation than angiogenesis. It has been recently demonstrated that proliferating cancer cells in oxic cancer zones have lower ^18^F-FDG uptake comparing to less proliferating cancer cells locating in hypoxic zones of a cancer [8–12], it is not surprise necrosis is associated with low ^18^F-FDG accumulation [11]. In a malignant cancerous lesion, areas of low ^18^F-FDG accumulation either represent well proliferating cancer cells or necrosis or non-cancerous stroma. In patient, high ^18^F-FDG accumulation does not mean the region with proliferating cancer cells, but indicates cancer cells are with low proliferating rate, lack of ^18^F-FDG does not necessarily mean absence of viable cancer cells. Accordingly, great attention should be paid to manage cancer therapy with ^18^F-FDG PET.

#### ^18^F-FDG uptake and glucose transporters

Glucose transportor-1 (GLUT-1) has been considered as an important factor for glucose metabolism, which over-expresses in hypoxic cancer cells. Clavo et al [22] found increased ^3^H-FDG uptake in carcinoma cell lines and attributed it partly due to increased membrane expression of GLUT-1. In contrast, Burgman et al [23] described hypoxia increased ^3^H-FDG uptake in MCF7 breast carcinoma cells even without any detectable increase in GLUT-1 expression, but an increase in glucose transporter activity. We found that metastatic peritoneal colorectal cancer cells manifest significant changes in ^18^F-FDG uptake when animals were switched breathing from air to carbogen even in the absence of apparent changes in GLUT-1 expression [12]. Although it is possible that alterations in hexokinase activity may be involved, Waki et al found no correlation between hexokinase activity and ^3^H-2-deoxyglucose uptake for different tumor cell lines [29]. High GLUT-1 expression by itself, therefore, is insufficient to ensure high ^18^F-FDG uptake [12]. Immunohistochemical assay of GLUT-1 expression has been used for evaluation of hypoxia fraction in patients, considering GLUT-1 as an “historic” hypoxia marker [12, 30], ^18^F-FDG PET may be more accurate than histological assay of GLUT-1 to estimate hypoxia fraction of malignancies in patients.

### Effect of high concentration oxygen breathing on ^18^F-FDG uptake

Li et al addressed the effect of carbogen breathing on ^18^F-FDG uptake in a dual-hypoxia markers studies, carbogen breathing significantly decreased hypoxia status in animal models of cancer [12, 31–35]. Carbogen-breathing producing rapid decrease in tumor ^18^F-FDG uptake raises concerns, in particular, interpretation difficulties may arise when the changes in ^18^F-FDG uptake are used to monitor the tumor's response to therapy; hypoxic status changes in the tumor induced by the therapy may be challenged when the patient becomes oxygen dependent. A pilot study confirmed that patients’ oxygen breathing during ^18^F-FDG uptake phase may blunt the degree of tumor ^18^F-FDG uptake measured by SUV_max_ [36].

### ^18^F-FDG uptake in metastases

We recently reported the use of correlative imaging methodologies to examine the uptake of ^18^F-FDG in disseminated peritoneal metastases growing in mice. Microscopic examination of peritoneal tumor sections indicated a characteristic relationship between the pattern of hypoxia and tumor size. In general, the smallest tumor deposits ( < ~1 mm diameter) showed intense hypoxic and relatively poorly perfused. Larger tumors (~1- 4 mm diameter) appeared relatively well perfused with higher proliferation rate and containing less hypoxia [9, 11, 12, 31, 37–39]. There was spatial co-localization between high levels of tumor ^18^F-FDG uptake, pimonidazole binding and GLUT-1 expression. Such regions tended to correspond to low levels of cellular proliferation and blood perfusion. In particular, the smallest hypoxic tumor deposits had highest ^18^F-FDG uptake. Less hypoxic larger tumors displayed relatively low ^18^F-FDG uptake [38]. Therefore, ^18^F-FDG uptake was significantly high in hypoxic microscopic tumors, and in only hypoxic regions of macroscopic tumors.

On microscopic examination, HT29 ascites tumors appeared similar to small avascular HT29 peritoneal tumors of < 1 mm in diameter. Ascites tumors are severely hypoxic [9, 11, 12, 31] which had high ^18^F-FDG uptake [12]. Therefore, in clinical setting, a PET/CT finding of diffusely increased ^18^F-FDG activity in ascites fluid may be an indicative of malignancies.

### Revisit ^18^F-FDG PET/CT application in oncology

**Figure 2:**
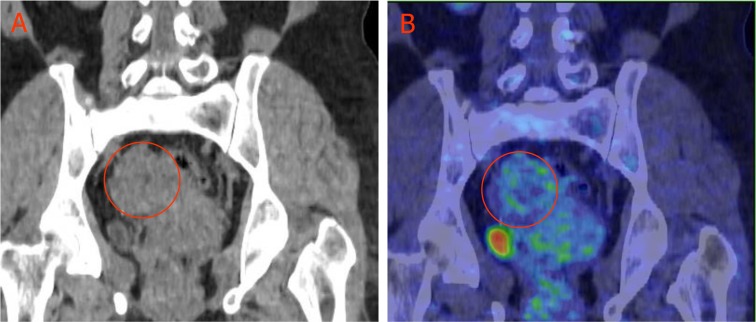
Low ^18^F-FDG uptake in ovary granulosa cell tumor **A** 54-yr-old female with pathologically confirmed granulosa cell tumor of the right ovary (circled), ^18^F-FDG accumulation in the tumor is low. A: CT imaging; **B**: ^18^F-FDG PET/CT overlay obtained 60 min after 5 mCi (185MBq) ^18^F-FDG intravenous injection.

During the past decades, the wide use of ^18^F-FDG PET and PET/CT in cancer detection demonstrated that some untreated malignant lesions (cancer type dependent) may have negative or low ^18^F-FDG uptake [15] (Figure 2). Ours and others preclinical research demonstrated a close ties between tumor ^18^F-FDG activity and its hypoxia. Thus, well oxygenated malignant tumors may be non-^18^F-FDG-avid, a combination of early phase perfusion and balance phase metabolism ^18^F-FDG PET imaging may give a more accurate estimation of viable cancer cell burden.

## CONCLUSIONS

^18^F-FDG highly accumulation in hypoxic and poorly proliferating cancer cells, but the uptake is low in oxic and well proliferating cancer cells. One should keep in mind that ^18^F-FDG is not a specific cancer-avid PET tracer, low ^18^F-FDG accumulation following anticancer therapy does not necessarily mean all cancer cells are eliminated; both well oxygenated cancer cells and necrosis associated with low ^18^F-FDG uptake. The successful application of ^18^F-FDG PET for cancer detection is because 95% of solid malignancies contain some degree of hypoxia [40–42]. Better understanding of the intratumoral ^18^F-FDG distributions will enable us to overcome most of the challenges that we face in our daily diagnostic and therapeutic clinical dilemmas in oncology, as well as systems molecular imaging application [43].

## MATERIALS AND METHODS

In this article, part of the data presented here was obtained from our laboratories and PET center. The animal protocols were approved by the Institutional Animal Care and Use Committees of Harbin Medical University and University of Louisville; The Human PET study the use of the human data was approved by institutional review board of Harbin Medical University, in all patients, a written content was obtained, and good clinical practice was followed. We systemic review up-to-date literature with respect to ^18^F-FDG, cancer, hypoxia, Warburg effect, and glucose metabolism in pubmed database.

### Cancer cell lines and nude mice

The human cancer H460, HT29 and A549 cells were used in experiments to generate xenografts in nude mice. All experiments were performed using 7 week old female nude mice. Animals were housed five *per* cage and kept in the institutional small animal facility at a constant temperature and humidity.

### Preparation of ^18^F-FDG, markers of hypoxia and perfusion

^18^F-fluoride was produced by an in-house cyclotron. ^18^F-FDG was prepared with an automated modular-lab system (Eckert & Ziegler; Berlin, Germany). Radiochemical yields by thin layer chromatography were approximately 75% and radiochemical purity was greater than 99%.

In animal studies, the hypoxia marker, pimonidazole hydrochloride (Hypoxyprobe Inc) was dissolved in physiological saline at a concentration of 20 mg/ml. The blood perfusion marker, Hoechst 33342 (Sigma-Aldrich) was dissolved in physiological saline at a concentration of 5 mg/ml. Fresh drug solutions were prepared on the day of each experiment. All injections were given *via* tail veins. Pimonidazole was given as 2 mg in 0.1 ml PBS; Hoechst 33342 as 0.5 mg in 0.1 ml PBS; 7.4 MBq of ^18^F-FDG was administered either singly (for PET imaging) or as a co-injection with pimonidazole. Hoechst 33342 was always administered 1minute before sacrifice. For patient studies, only ^18^F-FDG was administrated.

### Preparation of frozen tumor sections

Immediately after animal sacrifice, xenografts were removed, frozen and embedded in optimal cutting temperature medium (Sakura Finetek). Five contiguous 7-μm-thick tissue sections were cut using a Microm HM500 cryostat microtome (Microm International GmbH).

### Digital autoradiography

Autoradiograms were obtained by placing the tumor sections in a film cassette against an imaging plate as described previously [8]. The plate read by a Cyclone Plus imaging system (PerkinElmer, Inc). Regions of interest (100 × 100μm) were drawn over hypoxic cancer cells regions, oxic cancer cells regions and stroma in digital autoradiography referring immunohistochemical and hematoxylin and eosin stain images obtained from same or an adjacent section for digital autoradiography.

### Immunohistochemical staining of tumor sections

Pimonidazole and Hoechst 33342 images were obtained after completion of digital autoradiography exposures if applied. The same tumor sections were used for all images in order to minimize issues associated with section registration. Slides were air-dried, fixed in cold acetone (4°C) for 20 min, and incubated with SuperBlock (Pierce Biotechnology) at room temperature for 30 min. All antibodies were also applied in SuperBlock. Sections were then incubated with FITC conjugated anti-pimonidazole monoclonal antibody (Hypoxyprobe Inc) for one hour at room temperature.

### PET imaging

All animals were imaged in a prone position using Siemens Inveon Micro-PET (Siemens Medical Solutions Knoxville, TN) system. All human imaging was obtained from a GE DISCOVERY 690 ELITE PET/CT scanner.
